# Effectiveness of pharmacist intervention for deprescribing potentially inappropriate medications: a prospective observational study

**DOI:** 10.1186/s40780-022-00243-0

**Published:** 2022-04-05

**Authors:** Takeshi Kimura, Misa Fujita, Michiko Shimizu, Kasumi Sumiyoshi, Saho Bansho, Kazuhiro Yamamoto, Tomohiro Omura, Ikuko Yano

**Affiliations:** Department of Pharmacy, Kobe University Hospital, 7-5-2 Kusunoki-cho, Chuo-ku, Kobe, 650-0017 Japan

**Keywords:** Deprescribing, Potentially inappropriate medications, Polypharmacy, STOPP-J, STOPP criteria version 2

## Abstract

**Background:**

Potentially inappropriate medications (PIMs) and polypharmacy in older adults lead to increase the risk of adverse drug events. This study aimed to evaluate the effectiveness of pharmacist intervention combining the criteria for detecting PIMs with the deprescribing algorithm on correcting PIMs, reducing the number of medications, and readmissions.

**Methods:**

A prospective observational study was conducted at a Japanese University Hospital enrolling new inpatients aged ≥65 years prescribed ≥1 daily medication. Pharmacists detected PIMs based on the criteria combined the screening tool of older persons’ potentially inappropriate prescriptions criteria version 2 with the screening tool for older persons’ appropriate prescriptions for Japanese, examined changes using the deprescribing algorithm, and suggested changes to the physician. The proportion of patients whose number of medications was reduced at discharge and the rate of readmissions within 30 and 90 days were compared between patients without PIMs (without PIMs group), patients who were not suggested to change PIMs (no suggestions group), and patients who were suggested to change PIMs (suggested group).

**Results:**

The study enrolled 544 patients (median age 75.0 years, 54.4% males, median number of medications 6.0/patient). The number of patients with PIMs was 240 (44.1%), and 304 patients had no PIMs (without PIMs group). Among the patients with PIMs, 125 (52.1%) patients received pharmacist suggestions to change ≥1 PIMs (suggested group), and 115 patients received no suggestions for change (no suggestions group). The total number of PIMs was 432, of which changes were suggested for 189 (43.8%). Of these 189 cases, 172 (91.0%) were changed. The proportion of patients whose number of medications was reduced was significantly higher in the suggested group than in the without PIMs group and the no suggestions group [56.8% (71/125) vs. 26.6% (81/304) and 19.1% (22/115), respectively; *P* < 0.001 in both comparisons]. There were no significant differences in the rates of readmissions within 30 and 90 days among the three groups.

**Conclusions:**

Pharmacist intervention combining the criteria for detecting PIMs with the deprescribing algorithm was effective for correcting PIMs and may be associated with a reduction in the number of medications.

## Introduction

The global population has been aging, and this trend is particularly remarkable in developed countries [1]. Older adults often have multimorbidity, resulting in a state of polypharmacy. Inappropriate prescriptions and polypharmacy in older adults are associated with an increase in adverse drug events, drug-drug interactions, hospitalizations, medical resource utilization, healthcare costs, and mortality [2–8], which have been targeted for correction.

Cochrane review suggested that it is uncertain whether intervention to polypharmacy for older adults reduces the potentially inappropriate medications (PIMs) or patients’ clinical outcome [9]. Meanwhile, several randomized clinical trials have demonstrated the efficacy of pharmacist intervention on correcting PIMs [10], long-term discontinuations of PIMs [11], and reducing the number of medications prescribed [12]. As explicit criteria to screen for PIMs, the effectiveness of the screening tool of older persons’ potentially inappropriate prescriptions (STOPP)/screening tool to alert doctors to right treatment (START) criteria has been reported [13, 14]. As implicit criteria to reduce inappropriate polypharmacy, the deprescribing algorithm has been proposed [15]. Since the prevalence and type of PIMs vary by country and health care setting [16], corrective measures based on these variations should be developed in each country.

In Japan, polypharmacy in older adults has been a social problem as well [17–19], and incentives in medical fees have been paid for interventions to reduce the number of drugs in patients with polypharmacy. However, few studies have evaluated the effectiveness of intervention for PIMs in Japan [20, 21]. The screening tool for older persons’ appropriate prescriptions for Japanese (STOPP-J) has been published in 2016 by the Japan Geriatrics Society [22], and “List of drugs to be prescribed with special caution” in STOPP-J includes 29 criteria indicating PIMs [22]. We previously reported the effectiveness of pharmacist intervention using STOPP criteria version 2 (STOPP-v2) or STOPP-J in Japanese clinical settings [23, 24]. STOPP-J detected significantly more patients with PIMs than STOPP-v2 because of its wide applicability, although the number of changes in PIMs was comparable for both criteria [24]. STOPP-v2 and STOPP-J includes multiple criteria which targeted drugs are overlapped. Therefore, we hypothesized that the combination of STOPP-v2 and STOPP-J could detect PIMs in accordance with the prevalence of PIMs in Japan, and correct PIMs more efficiently. Moreover, previous our studies showed that there were many PIMs that could not change due to the risk of withdrawal symptoms or disease exacerbation [23, 24]. Combining the deprescribing algorithm [15] with the criteria for screening PIMs may assist in determining whether PIMs can be safely changed. In addition, it is necessary to evaluate whether pharmacist intervention for PIMs is effective in reducing the number of medications and improving clinical outcomes in order to resolve the problems of polypharmacy in Japan.

The objective of this study was to evaluate the effectiveness of pharmacist intervention combining the criteria for detecting PIMs with the deprescribing algorithm on reducing the number of medications and the rate of readmissions in a Japanese clinical setting.

## Methods

### Study design and settings

A prospective observational study was conducted from January to August 2018 at five medical units of Kobe University Hospital, Japan. The main departments in these units were General Internal Medicine, Neurology, Rheumatology and Clinical Immunology, Neurosurgery, Gastrointestinal Surgery, Cardiovascular Surgery, Cardiovascular Internal Medicine, Orthopaedic Surgery, and Breast Surgery. The study protocol was approved by the Ethical Committee of Kobe University Hospital (No. 1758), and the study was performed in accordance with the Declaration of Helsinki and its amendments. Informed consent was obtained in the form of opt-out on the website of the hospital. This report was followed the Strengthening the Reporting of Observational studies in Epidemiology (STROBE) statement [25].

### Detecting and changing PIMs by pharmacist intervention

**Fig. 1:**
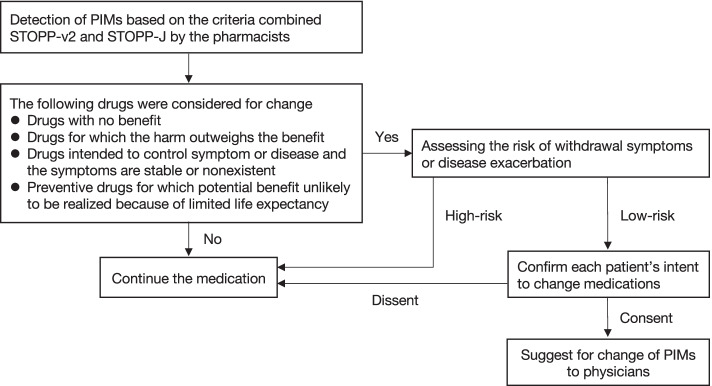
The scheme for detecting and changing PIMs. Abbreviations: PIMs, potentially inappropriate medications; STOPP-v2, Screening Tool of Older Persons’ potentially inappropriate Prescriptions criteria version 2; STOPP-J, Screening Tool for Older Persons’ appropriate Prescriptions for Japanese

Fifteen clinical pharmacists with 1 to 16 years of experience were participated in the study. Before participating in the study, they were trained to detect and change PIMs based on STOPP-v2 and STOPP-J. One or two of them worked in each subject unit on weekdays. Only oral medications were included for detecting and changing of PIMs, and external medications were not included in the study. The scheme for detecting and changing PIMs is shown Fig. 1. Namely, pharmacists conducted medication reconciliation, confirmed medical history and laboratory data, and detected PIMs at the time of patient admission. For the detection of PIMs, we used the criteria combined STOPP-v2 with STOPP-J. The pharmacists assessed whether the detected PIMs could be changed according to the deprescribing algorithm [12, 15]. The following drugs were considered for dose reduction, discontinuation, or change to other drugs if those risk of withdrawal symptoms or disease exacerbation was judged to be low: drugs with no benefit, drugs for which the harm outweighs the benefit, drugs intended to control symptom or disease and the symptoms are stable or nonexistent, and preventive drugs for which potential benefit unlikely to be realized because of limited life expectancy [15]. The pharmacists also confirmed each patient’s intent to change medications, and based on those intents and their assessments, suggested for discontinuation/change of PIMs to physicians. The pharmacists and the physicians discussed and arrived at a consensus regarding any changes.

STOPP-v2 consists of 80 criteria, each of which contains the drug class and indication [13]. Of the 29 criteria in STOPP-J, the drugs for 21 criteria overlap with STOPP-v2, and the drugs for other 8 criteria do not include in STOPP-v2. Accordingly, we combined the 8 criteria in STOPP-J with all 80 criteria in STOPP-v2, and used these combined 88 criteria in this study. In addition, we provided specific examples of the criterion for “any drug prescribed without an evidence-based clinical indication” in STOPP-v2. These examples of PIMs were detected in our previous studies [23, 24] and referred in the deprescribing algorithm [15], and are as follows: “concomitant use of proton-pump inhibitor (PPI) or H_2_ receptor antagonists and mucosal protective agent without a clinical indication,” “use of symptom control drug (such as antitussive agents or antiemetic drugs) when symptoms have already resolved,” and “use of vitamins in patients who have no clinical indication and can eat an adequate diet.”

### Sample size and study subjects

This study was conducted for a fixed period (8 months), and we assumed that 400–500 samples would be included during the study period. Patients aged ≥65 years who were newly admitted, had been in the hospital for ≥7 days, and were prescribed at least one daily medication were included. The main subjects of STOPP-J were individuals aged ≥75 years and older individuals who were not yet aged 75 years, but who were frail or in need of special care [22]. The targeted participants of this study were hospitalized patients, and based on the targeted age of STOPP-v2 [13], the subjects of this study were integrated to individuals aged ≥65 years. Since it was difficult to change medications in patients with short hospitalization in our previous studies [23, 24], we included patients who were hospitalized for ≥7 days in this study.

In order to assess the effectiveness of pharmacist intervention on deprescribing, we compared the proportion of patients whose total number of medications was reduced more than one at discharge compared with that at admission and the changes in the number of medications during the hospitalization between the following three groups: patients without PIMs (without PIMs group), patients who were not suggested to change PIMs by the pharmacists based on the deprescribing algorithm or patients’ dissent (no suggestions group), and patients who were suggested to change one or more PIMs by the pharmacists (suggested group). In addition, the rate of readmissions within 30 and 90 days in the three groups were evaluated as a clinical outcome for the patients. Readmissions included only unscheduled readmissions, and excluded scheduled admissions, such as for clinical examination, surgery, or chemotherapy. Patient characteristics (age, number of medications, and length of hospitalization) were also compared between the three groups. The number of PIMs, PIMs suggested changes, and PIMs changed before discharge were evaluated.

### Statistical analysis

The chi-square test or Fisher’s exact test followed by Bonferroni correction was used to compare the proportions of categorical variables between three groups (the proportion of patients whose total number of medications was reduced at discharge and the rate of readmissions within 30 and 90 days), and *P* values < 0.017 (0.05/3) were considered to indicate statistical significance. The statistical significance of the difference in median values between the three groups was analyzed by the Kruskal-Wallis test, followed by Dunn’s multiple comparisons test (age, number of medications, length of hospitalization, and changes in the number of medications during the hospitalization), and *P* values < 0.05 were considered to indicate statistical significance. All statistical analyses were performed with GraphPad Prism 6 (La Jolla, CA, USA).

## Results

### Patient characteristics

The characteristics of the study population are shown in Table 1. A total of 544 patients were included (median age 75.0 years, 296 [54.4%] males, median number of medications 6.0, median length of hospitalization 19.0 days). The number of patients with PIMs based on the criteria combined STOPP-v2 with STOPP-J was 240 (44.1%), and 304 patients had no PIMs (without PIMs group). Of the 240 patients with PIMs, 125 (52.1%) patients received pharmacist suggestions to change one or more PIMs (suggested group), and 115 patients received no suggestions for change (no suggestions group). The patients in the suggested group were significantly older than those in the without PIMs group (*P* = 0.029). Both patients in the suggested group and the no suggestions group had a higher number of medications than those in the without PIMs group (*P* < 0.001 and < 0.001). The patients in the suggested group had a longer length of hospitalization than those in the without PIMs group and the no suggestions group (*P* = 0.0029 and < 0.001). The most common department of all the study patients was Cardiovascular Surgery, followed by Orthopaedic Surgery.

**Table 1:** Characteristics of study population

		Total (*n* = 544)	Without PIMs group (*n* = 304)	No suggestions group (*n* = 115)	Suggested group (*n* = 125)
Male	*n* (%)	296 (54.4)	167 (54.9)	62 (53.9)	67 (53.6)
Age (years)	Median (IQR)	75.0 (70.0–80.0)	74.0 (69.0–79.0)	75.0 (69.0–80.0)	77.0 (71.0–81.0)^*^
Number of medications	Median (IQR)	6.0 (4.0–9.0)	5.0 (3.0–7.0)	7.0 (5.0–10.0) ^‡‡‡^	8.0 (6.0–11.0)^***^
Length of hospitalization (days)	Median (IQR)	19.0 (14.0–30.0)	19.0 (13.0–29.0)	18.0 (11.0–23.0)	25.0 (16.0–37.5)^**†††^
Departments					
Cardiovascular Surgery	n	185	115	32	38
Orthopaedic Surgery	n	107	52	31	24
Gastrointestinal Surgery	n	61	43	10	8
Neurology	n	55	29	10	16
Breast Surgery	n	47	22	12	13
Neurosurgery	n	46	22	7	17
Rheumatology and Clinical Immunology	n	31	14	9	8
Cardiovascular Internal Medicine	n	9	5	4	0
General Internal Medicine	n	3	2	0	1

*Abbreviations*: *IQR* interquartile range, *PIMs* potentially inappropriate medications

^*^P < 0.05

^**^P < 0.01

^***^P < 0.001 compared with the without PIMs group

^†††^*P* < 0.001 compared with the no suggestions group

^‡‡‡^*P* < 0.001 compared with the without PIMs group (Kruskal-Wallis test, followed by Dunn’s multiple comparisons test)

### Detected and corrected PIMs by pharmacist intervention

The number of each PIM and those changed after pharmacist intervention are shown in Table 2. The total number of PIMs based on the criteria combined STOPP-v2 with STOPP-J was 432. Of these, 189 (43.8%) were suggested for change by the pharmacists based on the deprescribing algorithm and patients’ consent, and 172 (91.0%) of whom were discontinued or changed after the pharmacist intervention. The most frequent PIMs identified was “Benzodiazepines for ≥ 4 weeks,” with 108 detected, 20 change suggestions, and 16 executed changes. The second most frequently identified PIMs was “Any drug prescribed without an evidence-based clinical indication,” with 84 detected, 75 change suggestions, and 67 executed changes. PIMs detected frequently in this criterion were “concomitant use of proton-pump inhibitor (PPI) or H_2_ receptor antagonists and mucosal protective agent without indication” and “use of vitamins in patients who have no indication and can eat an adequate diet,” and the number of detected, change suggestions, and executed changes were 26, 24, 18 and 29, 24, 20, respectively.

**Table 2:** Number of PIMs and those changed after pharmacist suggestion

Criteria^a^	Detected (*n* = 432)	Suggested (*n* = 189)	Changed (*n* = 172)
**STOPP-v2**	358	168	151
Drug indication criteria			
Any drug prescribed without an evidence-based clinical indication	84	75	67
Any duplicate drug class prescription	10	5	5
Cardiovascular System criteria			
Beta-blocker in combination with verapamil or diltiazem	1	0	0
Thiazide diuretic with current significant hypokalaemia, hyponatraemia, hypercalcaemia or with a history of gout	1	1	1
ACE inhibitors or Angiotensin Receptor Blockers in patients with hyperkalaemia	7	3	3
Coagulation System criteria			
Aspirin, clopidogrel, dipyridamole, vitamin K antagonists, direct thrombin inhibitors or factor Xa inhibitors with concurrent significant bleeding risk	1	1	1
Ticlopidine in any circumstances	4	3	3
NSAID and vitamin K antagonist, direct thrombin inhibitor or factor Xa inhibitors in combination	1	1	1
NSAID with concurrent antiplatelet agents without PPI prophylaxis	1	0	0
Central Nervous System criteria			
Benzodiazepines for ≥4 weeks^b^	108	20	16
Antipsychotics in those with parkinsonism or Lewy Body Disease	3	1	1
Anticholinergics/antimuscarinics in patients with delirium or dementia	5	4	3
First-generation antihistamines	3	3	3
Renal System criteria			
NSAIDs if eGFR < 50 mL/min/1.73m^2^	5	3	3
Gastrointestinal System criteria			
PPI for uncomplicated peptic ulcer disease or erosive peptic oesophagitis at full therapeutic dosage for > 8 weeks	28	12	11
Drugs likely to cause constipation in patients with chronic constipation where non-constipating alternatives are appropriate	3	1	0
Respiratory System criteria			
Benzodiazepines with acute or chronic respiratory failure	1	0	0
Musculoskeletal System criteria			
NSAID with established hypertension or heart failure	7	6	6
Long-term use of NSAID for symptom relief of osteoarthritis pain where paracetamol has not been tried	1	1	1
Long-term corticosteroids as monotherapy for rheumatoid arthritis	3	0	0
COX-2 selective NSAIDs with concurrent cardiovascular disease	3	2	2
NSAID with concurrent corticosteroids without PPI prophylaxis	1	0	0
Oral bisphosphonates in patients with a history of upper gastrointestinal disease	1	1	1
Urogenital System criteria			
Antimuscarinic drugs for overactive bladder syndrome with concurrent dementia or chronic cognitive impairment or narrow-angle glaucoma, or chronic prostatism	3	3	2
Endocrine System criteria			
Sulphonylureas with a long duration of action with type 2 diabetes mellitus	16	4	4
Beta-blockers in diabetes mellitus with frequent hypoglycaemic episodes	1	0	0
Drugs that predictably increase the risk of falls in older people			
Benzodiazepines	23	8	7
Vasodilator drugs with persistent postural hypotension	22	7	7
Hypnotic Z-drugs	9	2	2
Antimuscarinic/anticholinergic drug burden			
Concomitant use of two or more drugs with antimuscarinic/anticholinergic properties	2	1	1
**STOPP-J**	74	21	21
Sulpiride	3	1	1
H_2_ receptor antagonists	32	12	12
Laxative magnesium oxide (decreased kidney function)	23	8	8
α-glucosidase inhibitors	15	0	0
SGLT2 inhibitors	1	0	0

*Abbreviations*: *ACE* angiotensin-converting enzyme, *COX-2* cyclooxygenase-2, *eGFR* estimated glomerular filtration rate, *NSAID* non-steroidal anti-inflammatory drug, *PPI* proton-pump inhibitors, *SGLT2* sodium-glucose transporter 2, *STOPP-v2* Screening Tool of Older Persons’ potentially inappropriate Prescriptions criteria version 2, *STOPP-J* Screening Tool for Older Persons’ Appropriate Prescriptions for Japanese

^a^List of drugs includes only PIMs detected during the study period

^b^The criterion of “Benzodiazepines for ≥4 weeks” included both benzodiazepines and hypnotic Z-drugs

### Effectiveness of pharmacist intervention on reducing the total number of medications

**Fig. 2:**
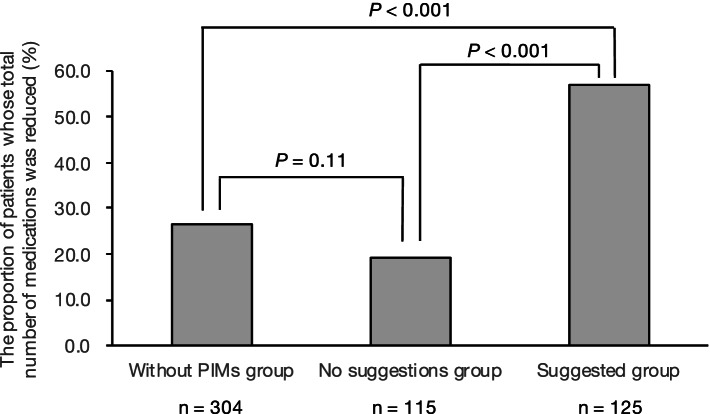
Proportion of patients whose total number of medications at discharge was reduced by more than one. The chi-square test followed by Bonferroni correction was used to compare the proportions of categorical variables between three groups, and *P* values < 0.017 were considered to indicate statistical significance. Abbreviations: PIMs, potentially inappropriate medications

**Fig. 3:**
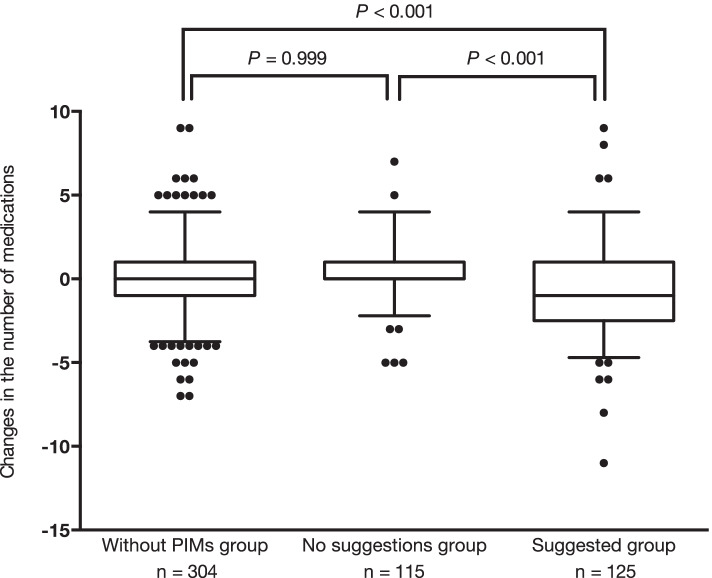
Changes in the number of medications during the hospitalization. Boxes represent interquartile ranges; whiskers, the 5th and 95th percentile in each category; dots mark outliers. Abbreviations: PIMs, potentially inappropriate medications

The proportion of patients whose total number of medications was reduced at discharge was 26.6% (81/304) in the without PIMs group, 19.1% (22/115) in the no suggestions group, and 56.8% (71/125) in the suggested group; the differences between the suggested group and the other groups were significant (*P* < 0.001 in both comparisons) (Fig. 2). The changes in the number of medications during the hospitalization decreased significantly in the suggested group than in the without PIMs group or the no suggestions group: median (interquartile range), − 1.0 (− 2.0 to 1.0) vs. 0.0 (− 1.0 to 1.0) and 0.0 (0.0 to 1.0); *P* < 0.001 in both comparisons (Fig. 3). The number of patients whose total number of medications was reduced more than two during the hospitalization were 43 (14.1%) in the without PIMs group, 11 (9.6%) in the no suggestions group, and 42 (33.6%) in the suggested group.

There were no significant differences in the rates of readmissions within 30 and 90 days among the three groups (Table 3).

**Table 3:** The rate of readmissions within 30 and 90 days

		Without PIMs group (*n* = 304)	No suggestions group (*n* = 115)	Suggested group (*n* = 125)	*P* values		
					Without PIMs group vs Suggested group	No suggestions group vs Suggested group	Without PIMs group vs No suggestions group
Readmissions within 30 days	*n* (%)	10 (3.3)	1 (0.9)	7 (5.6)	0.28	0.068	0.30
Readmissions within 90 days	*n* (%)	24 (7.9)	2 (1.7)	9 (7.2)	1.00	0.062	0.021

The Fisher’s exact test followed by Bonferroni correction was used to compare the proportions of categorical variables between three groups, and *P* values < 0.017 were considered to indicate statistical significance

*Abbreviations*: *PIMs* potentially inappropriate medications

## Discussion

In the present study, we used the criteria combined STOPP-v2 with STOPP-J, and suggested for a prescription change according to the deprescribing algorithm in patients with detected PIMs. Out of a total of 432 detected PIMs, 189 were suggested by pharmacists for change, and 91.0% of whom were changed. Furthermore, the suggested group showed a significantly higher proportion of patients whose total number of medications was reduced at discharge than the groups without PIMs or received no suggestions. No significant differences were observed in the rates of readmissions between the suggested group and the other two groups.

Our study suggested that pharmacist intervention was effective in correcting PIMs and may be associated with a reduction in the number of medications during the hospitalizations. No statistical sample size calculations were conducted before the start of the study. However, the power of the post-hoc analysis of the comparison between the suggested group and the other two groups for the proportion of patients whose total number of medications was reduced at discharge was 1.0 for both comparisons, thus we considered that the sample size of this study was sufficient. In Japan, medical fees began to be provided in 2016 for interventions to reduce the number of two or more drugs in patients with polypharmacy. More than 30% of patients in the suggested group had a reduction of two or more drugs in this study. A retrospective observational study using nationwide health insurance reimbursement claims data in Japan suggested that a 7.3% reduction in nationwide polypharmacy after the implementation of this health policy in 2016 [26]. On the other hand, there are few studies which evaluated the intervention for PIMs in Japan [20, 21]. Several randomized clinical trials in other countries have demonstrated the efficacy of pharmacist intervention on correcting PIMs [10–12], and our results were in line with these previous studies [10–12]. Meanwhile, this study did not show a reduction in readmissions by the pharmacist intervention for PIMs. Although the no suggestions group had a lower rate of readmissions than the other two groups, the differences were not statistically significant. In some cases, correcting PIMs was not suggested for patients with shorter hospitalization who were scheduled for readmission, thus the no suggestions group included more scheduled readmissions. Patients scheduled for readmission were less likely to be unscheduled readmission, which may have resulted in a lower rate of unscheduled readmissions in the no suggestions group. Cochrane review suggested that it is uncertain whether interventions for polypharmacy and medication reviews reduces hospital admissions, quality of life, or mortality [9, 27], and subsequent randomized clinical trial also did not demonstrate that clinical pharmacist intervention can reduce adverse drug-related incidents or clinically important medication errors during the posthospitalization [28]. Whereas, the other randomized clinical trial suggested that clinical pharmacist intervention including follow-up after discharge can reduce the number of emergency department visits and hospital readmissions [29]. In addition to intervention during hospitalization, post-discharge follow-up may be necessary to improve clinical outcome such as readmissions.

In this study, PIMs corresponded to “any drug prescribed without an evidence-based clinical indication” were most frequently changed. We described mucosal protective agents or vitamins as examples for this criterion, and these drugs were frequently detected as PIMs. Previous studies in Japan [18, 19, 23, 24] or outside of Japan [30, 31] have not been frequently detected these PIMs. However, our study suggested that these drugs may be abundantly prescribed in Japan. Although discontinuation of these drugs may not entirely contribute to improve clinical outcomes, it may lead to reduce unnecessary drug costs. PIMs related to benzodiazepines and PPI were frequently detected in this study, nevertheless the proportion of change of these drugs were low. Inappropriate prescribing of these drugs needs to be corrected in order to prevent adverse drug events in older patients. In this study, the pharmacists assessed whether PIMs could be safely changed according to the deprescribing algorithm, and suggested for those change based on the patients’ intents as well. Reasons for not suggesting changes of PIMs included that prescribed medications were needed for disease control and those benefits were high, the change was difficult due to the high risk of withdrawal symptoms, and lack of patient’s consent. The deprescribing algorithm was useful for pharmaceutical assessment. However, the combination of the other deprescribing algorithms by the Bruyère Research Institute [32, 33] may be effective for more specific assessment of prescription changes in each type of drug. In some cases where the scheduled duration of hospitalization was short, the pharmacists judged it too difficult to change PIMs before discharge and did not suggest those changes; thus, the length of hospitalization was longer in the suggested change group than the no suggestions group. Discontinuation of drugs with risk of withdrawal symptoms, such as benzodiazepines, is difficult in short hospitalization. Consequently, collaboration with community pharmacies, as in previous study [11], would be necessary for long-term changes of those PIMs.

Among the eight criteria unique to STOPP-J, H_2_ receptor antagonists and laxative magnesium oxide were frequently detected as PIMs. These PIMs were also detected in previous study in Japan [18, 24], and should be corrected, especially in patients with chronic kidney disease or those who have suffered an adverse event. Meanwhile, out of a total of 88 criteria combining the STOPP-v2 and STOPP-J in this study, PIMs were actually detected in 35 criteria. The contents of PIMs were similar in our previous studies [23, 24]. In order to detect PIMs more efficiently, it may be better to narrow down the contents of the criteria for the detection of PIMs.

This study has several limitations. First, it was an observational study, and we could not compare the changes in the number of medications during hospitalization between patients whose pharmacist suggested changes in PIMs and the entire population of patients with PIMs for whom no pharmacist intervention. Although there may be an association between pharmacist intervention for patients with PIMs and a reduction in their number of medications, a design that accurately evaluates the effect of pharmacist intervention is needed. Second, this study conducted at a single Japanese university hospital. Thus, our results may not be generalizable to other clinical settings or countries. However, our results may be useful in an aging population like Japan or clinical settings where prescribing trends are similar.

## Conclusion

The present study suggested the effectiveness of pharmacist intervention combining the criteria for detecting PIMs with the deprescribing algorithm on correcting PIMs and those association with a reduction the total number of medications at a discharge. Pharmacist intervention could correct most of the PIMs that were judged to be needed for change, and may be effective in reducing the number of medications during the hospitalizations, whereas did not demonstrate a reduction in readmissions. Pharmacist intervention could be useful in Japanese clinical settings, where PIMs and polypharmacy has been an urgent problem.

## Data Availability

The datasets used and/or analyzed during the current study are available from the corresponding author on reasonable request.

## Abbreviations

ACE: Angiotensin-converting enzyme
COX-2: Cyclooxygenase-2
eGFR: Estimated glomerular filtration rate
NSAID: Non-steroidal anti-inflammatory drug
PIMs: Potentially inappropriate medications
PPI: Proton-pump inhibitor
SGLT2: Sodium-glucose transporter 2
START: Screening Tool to Alert doctors to Right Treatment
STOPP: Screening Tool of Older Persons’ potentially inappropriate Prescriptions
STOPP-J: Screening Tool for Older Persons’ Appropriate Prescriptions for Japanese
STOPP-v2: Screening Tool of Older Persons’ potentially inappropriate Prescriptions criteria version 2
STROBE: Strengthening the Reporting of Observational studies in Epidemiology
